# *TET2* Overexpression in Chronic Lymphocytic Leukemia Is Unrelated to the Presence of *TET2* Variations

**DOI:** 10.1155/2014/814294

**Published:** 2014-02-18

**Authors:** María Hernández-Sánchez, Ana Eugenia Rodríguez, Alexander Kohlmann, Rocío Benito, Juan Luis García, Alberto Risueño, Encarna Fermiñán, Javier De Las Rivas, Marcos González, Jesús-María Hernández-Rivas

**Affiliations:** ^1^IBSAL, IBMCC, Centro de Investigación del Cáncer, Universidad de Salamanca-CSIC, 37007 Salamanca, Spain; ^2^Department of Molecular Genetics, MLL Munich Leukemia Laboratory, 81377 Munich, Germany; ^3^Instituto de Estudios de Ciencias de la Salud de Castilla y León (IECSCYL) and Hospital Clínico Universitario de Salamanca (HUSAL), 37007 Castilla y León, Spain; ^4^Celgene Institute for Translational Research Europe (CITRE), 41092 Sevilla, Spain; ^5^Unidad de Bioinformática y Genómica Funcional, IBMCC, Centro de Investigación del Cáncer, Universidad de Salamanca-CSIC, 37007 Salamanca, Spain; ^6^Unidad de Genómica, IBMCC, Centro de Investigación del Cáncer, Universidad de Salamanca-CSIC, 37007 Salamanca, Spain; ^7^Servicio de Hematología y Departamento de Medicina, Hospital Clínico Universitario de Salamanca, Paseo San Vicente 58, 37007 Salamanca, Spain

## Abstract

*TET2* is involved in a variety of hematopoietic malignancies, mainly in myeloid malignancies. Most mutations of *TET2* have been identified in myeloid disorders, but some have also recently been described in mature lymphoid neoplasms. In contrast to the large amount of data about mutations of *TET2*, some data are available for gene expression. Moreover, the role of TET2 in chronic lymphocytic leukemia (CLL) is unknown. This study analyzes both *TET2* expression and mutations in 48 CLL patients. *TET2* expression was analyzed by exon arrays and quantitative real-time polymerase chain reaction (qRT-PCR). Next-generation sequencing (NGS) technology was applied to investigate the presence of *TET2* variations. Overexpression of *TET2* was observed in B-cell lymphocytes from CLL patients compared with healthy donors (*P* = 0.004). In addition, in CLL patients, an overexpression of *TET2* was also observed in the clonal B cells compared with the nontumoral cells (*P* = 0.002). However, no novel mutations were observed. Therefore, overexpression of *TET2* in CLL seems to be unrelated to the presence of genomic *TET2* variations.

## 1. Introduction

The Ten-Eleven-Translocation 2 (*TET2*) gene encoding a 2-oxoglutarate/Fe2+ oxygenase catalyses mainly the conversion of methylcytosine to hydroxymethylcytosine. *TET2* is implicated in a variety of hematopoietic malignancies, particularly myeloid malignancies. Mutations of *TET2* have recently been identified in 12–26% in MDS/MPN disorders [1–5], 8–19% of adult acute myeloid leukemias (AML) [6, 7]. Its highest incidence has been found in chronic myelomonocytic leukemia (CMML) patients (50%) [8]. However, the presence of somatic mutations of the *TET2* gene in human mature lymphoid neoplasms has been recently described, whereby *TET2* mutations were present in 2–12% of B-cell and 12% of T-cell neoplasms [9–11].

In contrast to the considerable information about mutations of *TET2*, data about expression of this gene are scarce. *TET2* showed a broad expression pattern in different tissues in healthy donors, highlighting a 10- to 100-fold higher expression in hematological cells, the highest values being seen in granulocytes. In addition, *TET2* expression was lower in the granulocytes from MDS cases compared with healthy donors, irrespective of the *TET2* mutation status [3].

*TET*2 expression and mutations have been less studied in chronic lymphocytic leukemia (CLL) and only rare cases of this disease showed *TET2* mutations [12]. In the present study overexpression in CLL patients compared with healthy donors was observed. In addition, Next-generation sequencing studies confirmed that the presence of *TET2* mutations is rare in CLL.

## 2. Material and Methods

### 2.1. Patients

In total, 48 samples from CLL patients at diagnosis and 6 healthy donors were analyzed. CLL diagnosis was performed according to the World Health Organization (WHO) classification [13] and Working Group of National Cancer Institute (NCI) criteria [14]. In all cases, a complete immunophenotypic analysis by flow cytometry [15] and FISH studies were carried out. Main biological features of the 48 CLL patients included in the study are shown in Table 1. The study was approved by the local ethical committees “Comité Ético de Investigación Clínica, Hospital Universitario de Salamanca.” Written informed consent was obtained from each patient before they entered the study.

Both CLL B lymphocytes and normal B lymphocytes were purified using magnetically activated cell sorting (MACS) CD19 MicroBeads (Miltenyi Biotec, Bergisch Gladbach, Germany). CD19 selection resulted in >98% purity, as analyzed by flow cytometry.

Genomic DNA and total RNA were obtained from clonal B-cell lymphocytes (CD19+ cell fraction) and the remaining cells (CD19− cell fraction). DNA was isolated by QIAgen (Qiagen, Valencia, CA, USA), following the manufacturer's recommendations. RNA isolation was carried out using TRIZOL reagent.

### 2.2. Expression Analysis

Genome-wide expression analysis of the isolated samples of 27 CLL patients and 5 healthy donors was performed using Human Exon 1.0 microarrays (Affymetrix, Inc., Santa Clara, CA, USA) following the manufacturer's protocols for the GeneChip platform by Affymetrix, as previously reported [16]. Hybridized Affymetrix arrays were scanned with an Affymetrix GeneChip 3 000 scanner. Image generation and feature extraction were performed using Affymetrix GCOS Software.

SYBR Green quantitative Real-Time PCR was done in triplicate with SYBR Green mix (Applied Biosystems, Foster City, CA) using the IQ5 Multicolor Real-Time PCR Detection System (Bio-Rad) in a subset of CLL patients (*n* = 23) and healthy donors (*n* = 5) with the following gene-specific primers: *GAPDH*, forward 5′-CAGGGCTGCTTTTAACTCTGG-3′ and reverse 5′-GGGTGGAATCATATTGGAACA-3′, and *TET2*, forward 5′-GGGTGGAATCATATTGGAACA-3′ and reverse 5′-TGGACACAACCACAAATTCA-3′. The *GAPDH *gene was used as the internal control and the quantification of relative expression (reported as arbitrary units (a.u.)) were performed using the comparative Ct method. For analytical purposes, cut-off values were adopted according to median-expression levels for *TET2*.

### 2.3. Next-Generation Sequencing

NGS was carried out using the Roche GS FLX Titanium sequencing platform to investigate the *TET2* mutations in 26 CLL patients. First, NimbleGen Sequence Capture was applied to sequence the entire *TET2* gene (*n* = 4) with a median coverage more than 20X. Then, the 27 amplicons of the complete *TET2* coding region (*n* = 24) were sequenced using 454 FLX amplicon deep-sequencing chemistry with a median coverage of 689 reads, as previously described [17, 18]. *TET2* was sequenced by both strategies in 2 patients. Moreover, the gene expression profile of 5 out of 26 CLL patients was also analyzed by expression arrays.

Sanger sequencing was performed in CD19-positive and -negative cell fractions. Moreover, in 6 healthy donors a region of exon 11 was sequenced to analyze the association of one polymorphism in CLL. Primers used for Sanger sequencing are shown in Table 2.

### 2.4. Bioinformatic Analysis

For the exon array analysis, the robust microarray analysis (RMA) algorithm was used for background correction, intra- and intermicroarray normalization, and expression signal calculation [19]. Significance analysis of microarray (SAM) [20] was used to calculate significant differential expression. All bioinformatic analyses were performed with the statistical program R, as previously described [21].

The expression data from quantitative SYBR Green PCR were not normally distributed, so nonparametric tests were used. Expression levels of *TET2* in the different groups were analyzed using the Mann-Whitney test with a two-tailed value of *P* < 0.05 taken as indicating statistical significance. All tests were performed using SPSS v19.0.

Sequencing data from the Sequence Capture experiments were analyzed using GS Run Browser and GS Reference Mapper software, version 2.0.01 (Roche Diagnostics, Mannheim, Germany). All putative variants were compared with published single-nucleotide polymorphism (SNP) data (dbSNP build 130).

Amplicon deep-sequencing data were generated using GS FLX Sequencer Instrument, version 2.3, and analyzed with GS Amplicon Variant Analyzer, version 2.3 (Roche Diagnostics). The results were further processed and visualized following a previously described pipeline [17].

## 3. Results and Discussion

### 3.1. Overexpression of *TET2* in B Cells of CLL Compared with Healthy Donors

The expression levels of *TET2* were analyzed in CLL patients (*n* = 27) and healthy donors (*n* = 5) using oligonucleotide microarrays. Overexpression of the mRNA levels of *TET2* in CLL patients compared with healthy donors was observed (*P* = 0.004).

**Figure 1:**
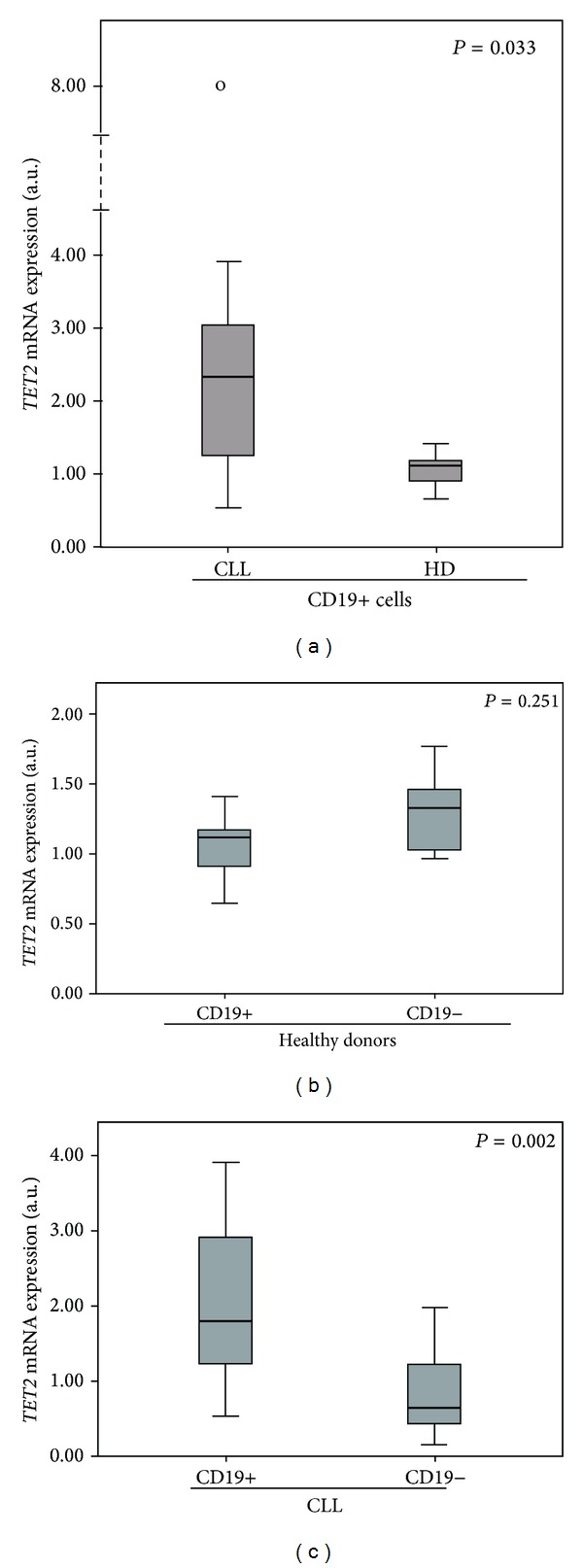
(a) Expression of *TET2* in B cells of CLL patients compared with healthy donors (HD). Box plot of the expression levels represented as arbitrary units (a.u.) of *TET2* showing significantly different levels of expression between CLL patients (*n* = 23) and healthy donors (HD) (*n* = 5), assessed by qRT-PCR. They indicate the overexpression of *TET2* in B cells (CD19+ cells) of CLL patients compared with healthy donors (*P* < 0.05). The thick line inside the box plot indicates the median expression levels and the box shows the 25th and 75th percentiles, while the whiskers show the maximum and minimum values. Outliers are represented by open circles. (b) Expression of *TET2* in CD19+ and CD19− cells of healthy donors. It shows a tendency towards overexpression of *TET2* in CD19− cells compared with CD19+ cells in healthy donors (*n* = 5) (*P* > 0.05). (c) Expression of *TET2* in CD19+ and CD19− cells of CLL patients. It illustrates the overexpression of *TET2* in B clonal cells (CD19+ cells) compared with normal cells (CD19− cells) in CLL patients (*n* = 15) (*P* < 0.05).

These results were confirmed in 23 CLL patients and 5 healthy donors by qRT-PCR. Clonal B cells from CLL patients had a significantly higher expression of *TET2* than B cells from healthy donors (*P* = 0.033) (Figure 1(a)).

In myeloid malignancies, *TET2* expression has been shown to be lower in MDS cases than in healthy controls, irrespective of the *TET2* mutation status [3]. However, there have been no studies addressing the expression levels of *TET2* in CLL. TET2 plays an important role in the metabolism of 5-methylcytosine to 5-hydroxymethylcytosine (5-hmC) [22]. In addition, inactivation of *TET2* is related to low levels of 5-hmC, as it has been reported in HEK293T cells [23]. In the opposite way, *TET2* overexpression could lead to an increase of 5 hmC in CLL patients.

Moreover, *TET2* overexpression in CLL was not associated with other biological markers with well-known prognostic value in CLL such as *IGHV* mutational status (*P* = 0.67) or cytogenetic alterations (*P* = 0.197).

### 3.2. Differences in *TET2* Expression in B Clonal Cells and Normal Cells in CLL Patients

The expression differences between the B-cell lymphocytes (CD19+) and the remaining cells (CD19−) were studied. In healthy donors (*n* = 5), CD19− cells had a higher level of *TET2* expression than CD19+ cells (Figure 1(b)), consistent with the previously described highest expression of *TET2* in granulocytes [3]. As expected, *TET2* expression depends on the cell type [3]. By contrast, overexpression was observed in the clonal CD19+ cells of CLL patients compared with CD19− cells (*P* = 0.002) (Figure 1(c)). To our knowledge, such results have not been previously reported.

*TET2* overexpression in CLL patients could be explained by the deregulation of expression of other members of the TET family in B cells. Compensatory action in the TET family has also been suggested by a previous study [24]. However, a *TET2* deficient murine model has recently been proposed in which the loss of *TET2* during adult hematopoiesis is not compensated by increased transcription of *TET1* and *TET3 *[9].

### 3.3. *TET2* Overexpression Is Unrelated to the Presence of the Gene Mutations

To determine whether *TET2* overexpression could be related to the presence of mutations or polymorphisms in *TET2*, sequencing studies were carried out. No novel variants in *TET2* were revealed from the deep sequencing study following the Sequence Capture strategy.

To further assess the presence and prevalence of *TET2* mutations in CLL, the *TET2* coding sequence was also analyzed in 24 CLL patients by 454 amplicon deep-sequencing. Sequencing data of all coding exons of *TET2*, represented by 27 amplicons, confirmed the presence of known polymorphisms in *TET2*. However, we did not detect any mutations that might affect the protein.

Therefore, NGS did not enable any novel and relevant mutations in *TET2* in CLL patients to be detected. Our sequencing results are consistent with the data provided by the International Cancer Genome Consortium and those recently published in which the *TET2* mutations were rarely present in CLL [12, 25]. Thus, the overexpression of *TET2* in chronic lymphocytic leukemia was unrelated to the presence of *TET2* mutations.

However, a large number of single nucleotide variations described in the SNP database were detected, confirming that the *TET2* gene is a polymorphic gene. Most of these polymorphisms were localized in exons 3 and 11 (Table 3). Due to the primers used in NGS, three variations were also detected in noncoding regions near the analyzed exons. One of these polymorphisms, rs2454206, situated in exon 11, was found in 54% (14/26) of CLL patients. To determine whether this polymorphism could be associated with CLL, 6 healthy donors were sequenced by Sanger. The same polymorphisms were found in 83% (5/6) of these cases.

Recently, the correlation between the presence of polymorphisms in *IDH1* gene and the overexpression of the gene has been described in MDS [26]. To determine whether the presence of *TET2* polymorphisms could influence the overexpression of this gene, the association between gene expression and genetic data was investigated. The expression was analyzed in the clonal CD19+ cells of CLL patients showing polymorphisms (*n* = 16) and in CLL patients without polymorphisms (*n* = 2). The results showed similar *TET2* expression levels in both CLL groups (data not shown).

NGS analysis of the 15 patients analyzed by qRT-PCR showed that all but one had polymorphisms in TET2 in both CD19+ and CD19− cells. The clonal CD19+ lymphocytes of CLL patients showing polymorphisms (*n* = 14) were also compared with corresponding CD19− cells. As expected, the tumoral cell fraction of these cases was more strongly expressed (*P* = 0.001) than the nontumoral cell fraction (data not shown). Thus, *TET2* expression could not be related to the presence of polymorphisms and the functional effects of harboring these polymorphisms are unclear.

In conclusion, *TET2* overexpression in CLL patients relative to healthy donors and overexpression of *TET2* in B clonal cells compared with nonclonal B cells in CLL patients have been demonstrated. In addition, *TET2* overexpression is unrelated to the presence of the gene mutations and is independent of the presence of polymorphisms.

The relevance of *TET2* overexpression in CLL remains unknown. Given the role of TET2 in the conversion of methylcytosine to hydroxymethylcytosine, it could be related to epigenetic mechanisms.

## 4. Conclusions

To the best of our knowledge, this is the first study about the role of *TET2* in CLL: this gene was overexpressed in clonal B cells in CLL. However the overexpression of *TET2* in CLL is not related to *TET2* variations. This report suggested that not only loss of function but also hyperfunction of *TET2* could be related to tumorigenesis.

## Figures and Tables

**Table 1:** Characteristics of the 48 CLL patients included in the study.

Parameter	Category	%
Age (years), median (range)		65 (37–84)
Gender	Male	54
White blood cells (×109/L), median (range)		24,980 (10,130–96,510)
Lymphocytes (×109/L), median (range)		21,070 (5,900–86,320)
Hemoglobin (×109/L), median (range)		13.6 (11.5–15.8)
Platelet (×109/L), median (range)		176,500 (70,000–333,000)
Binet stage	A	67
	B	27
	C	6
Lactate dehydrogenase	High	7
*β*2 microglobulin	High	25
*IGHV *	Unmutated	45
FISH abnormality*	13q deletion	56
	11q deletion	15
	17p deletion	2
	Trisomy 12	17
	No FISH abnormalities	29

*Some patients have more than one cytogenetic aberration.

**Table 2:** Sequences of primers used for Sanger sequencing.

Primer designation	Sequence (5′-3′)
*TET2* exon 3 (ENSE00002471221)	
Forward	GGAACACACACATGGTGAAC
Reverse	TGGAACAGTCATTGTCCCTG

*TET2* exon 11 (ENSE00002415078)	
Forward	TCCCATGAACCCTTACCCTG
Reverse	ACGCTTTGCACACTCAATG

**Table 3:** Known variations of *TET2* detected in 26 CLL cases by next-generation sequencing.

SNP	rs	Region	Protein	*n* (%)
86C>G	rs12498609	exon 3	Pro29Arg	3 (12)
100C>T	rs111948941	exon 3	Leu34Phe	2 (8)
521C>A	rs146031219	exon 3	Pro174His	1 (4)
652G>A	rs6843141	exon 3	Val218Met	2 (8)
1088C>T	rs17253672	exon 3	Pro363Leu	2 (8)
1105C>T	rs150072691	exon 3	Arg369Trp	1 (4)
2599T>C	rs144386291	exon 3	Tyr867His	1 (4)
5162T>G	rs34402524	exon 11	Leu1721Trp	4 (15)
5167C>T	rs146348065	exon 11	Pro1723Ser	1 (4)
5284A>G	rs2454206	exon 11	Ile1762Val	14 (54)
5333A>G	rs62621450	exon 11	His1778Arg	3 (12)

3803 + 45G>A	rs17319679	Intron 7	—	2 (8)
4045 − 35C>A	rs59519484	Intron 8	—	1 (4)
*74G>A	rs60786079	3′UTR	—	3 (12)

*N = nucleotide N 3′ of the translation stop codon.
